# Neglected trauma-induced testicular torsion: Clinical, sonographic, and surgical correlation in a 13-year-old adolescent

**DOI:** 10.1016/j.radcr.2023.07.008

**Published:** 2023-07-22

**Authors:** Manar Ezzahi, Sara El Malih, Zaid Ennasery, Naoufal Boumahdi, Amal Akammar, Nizar El Bouardi, Meriem Haloua, Badreeddine Alami, Moulay Youssef Alaoui Lamrani, Mustapha Maaroufi, Youssef Bouabdallah, Meryem Boubbou

**Affiliations:** a Department of Mother and Child Radiology, CHU Hassan II Fez, Sidi Mohammed Ben Abdellah University, Fez, Morocco; b Department of Adult Radiology, CHU Hassan II Fez, Sidi Mohammed Ben Abdellah University, Fez, Morocco; c Department of Pediatric Surgery, CHU Hassan II Fez, Sidi Mohammed Ben Abdellah University, Fez, Morocco

**Keywords:** Testicular, Torsion, Traumatic, Necrotic, Orchidectomy

## Abstract

Post-traumatic testicular torsion is a rare condition, accounting for approximately 4%-8% of all reported cases of testicular torsion in the literature. Prompt clinical suspicion and intervention are crucial; as testicular torsion is considered a medical and surgical emergency that may lead to testicular necrosis. Ultrasound imaging plays an important role in assessing the integrity of the traumatized scrotum and facilitating early detection of associated testicular torsion. In this case report, we present a neglected post-traumatic testicular torsion in a 13-year-old child that led to orchiectomy.

## Introduction

Testicular torsion is by far the most prevalent urological emergency before the age of 25. Trauma is an underappreciated cause of this pathology [1,2].

It occurs notably when predisposing anatomical factors are present, such as “bell clapper deformities” [3].

A traumatized testicle is often swollen, making a meticulous examination difficult, and symptoms can easily be mistakenly attributed to the trauma than torsion.

Clinical examination findings vary with the duration and degree of spermatic cord rotation. Ultrasound imaging plays a crucial role in assessing the integrity of the contents of the traumatized scrotum. This evaluation is essential for the early detection of traumatic testicular torsion and the identification of any associated complications [4].

An early diagnosis can ensure testicular salvage and help avoid unnecessary complications such as testicle necrosis.

In this case report, we present a case of post-traumatic testicular torsion in a 13-year-old boy. We will discuss the diagnosis, treatment, and prognosis of this condition based on a comprehensive review of the existing literature.

## Case report

We report the case of a 13-year-old male child admitted to the pediatric emergency room 32 hours after testicular trauma following a bicycle accident. The patient initially received analgesic treatment in the first few hours following the trauma. However, due to the persistence of pain and an increase in scrotal swelling, the patient sought medical attention on the second day.

**Fig. 1:**
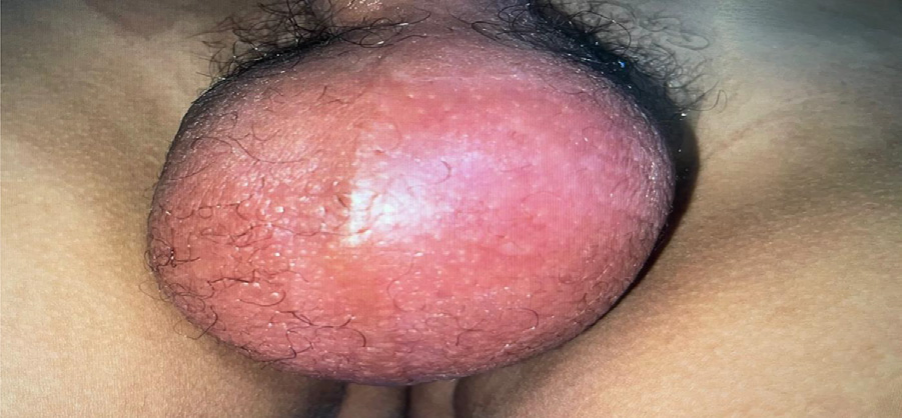
Clinical picture on patient admission: bursa increased in volume and erythematous.

Clinical examination revealed a large, painful, reddish bursa (Fig. 1), very tender on palpation, with a negative “Prehn” sign, a positive “Governor” sign, and an abolished cremasteric reflex on the left side.

An ultrasound exploration was immediately requested by the surgeon to explore the scrotal content.

**Fig. 2:**
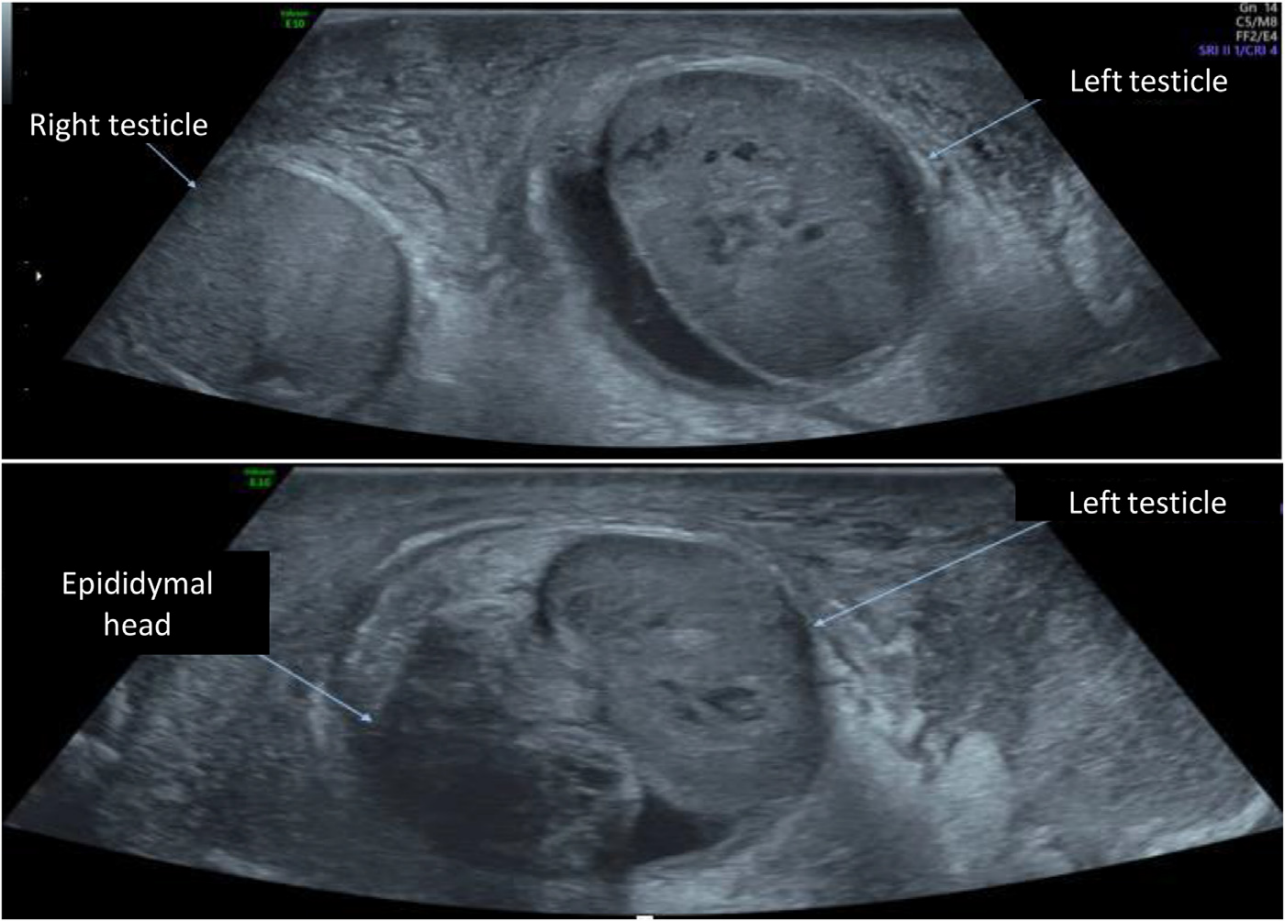
Study of the testicles in mode B: ascended left testicle, which is heterogeneous and hypoechoic with visualization of very hypoechoic areas within it. We also note a swollen and heterogeneous appearance of the head of left epididymis, as well as a left low abundance hematocele.

**Fig. 3:**
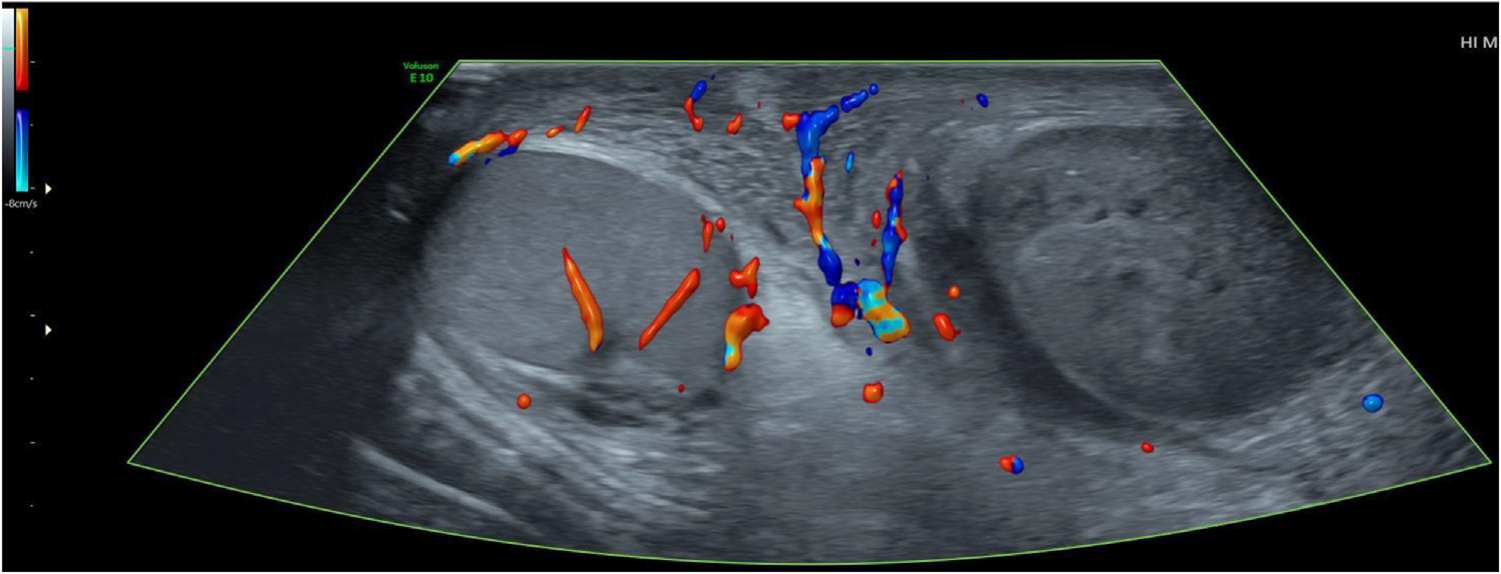
Comparative color Doppler study of the testicles: demonstrates the absence of vascularization of the left testicle. We also note the hyperhemia and thickening of scrotal wall.

**Fig. 4:**
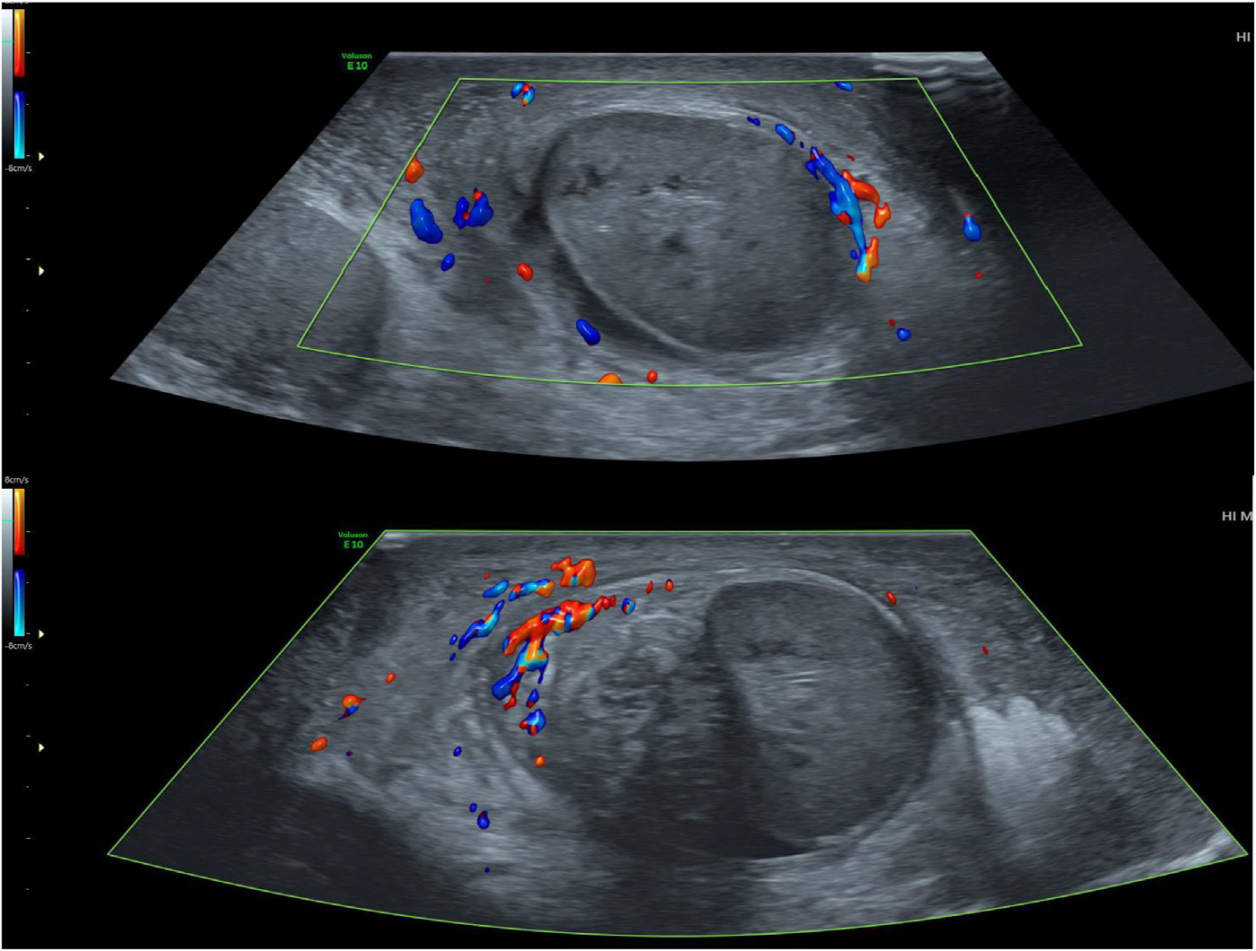
Color Doppler study of the left testicle, objectifying the absence of vascularization of the testicle.

**Fig. 5:**
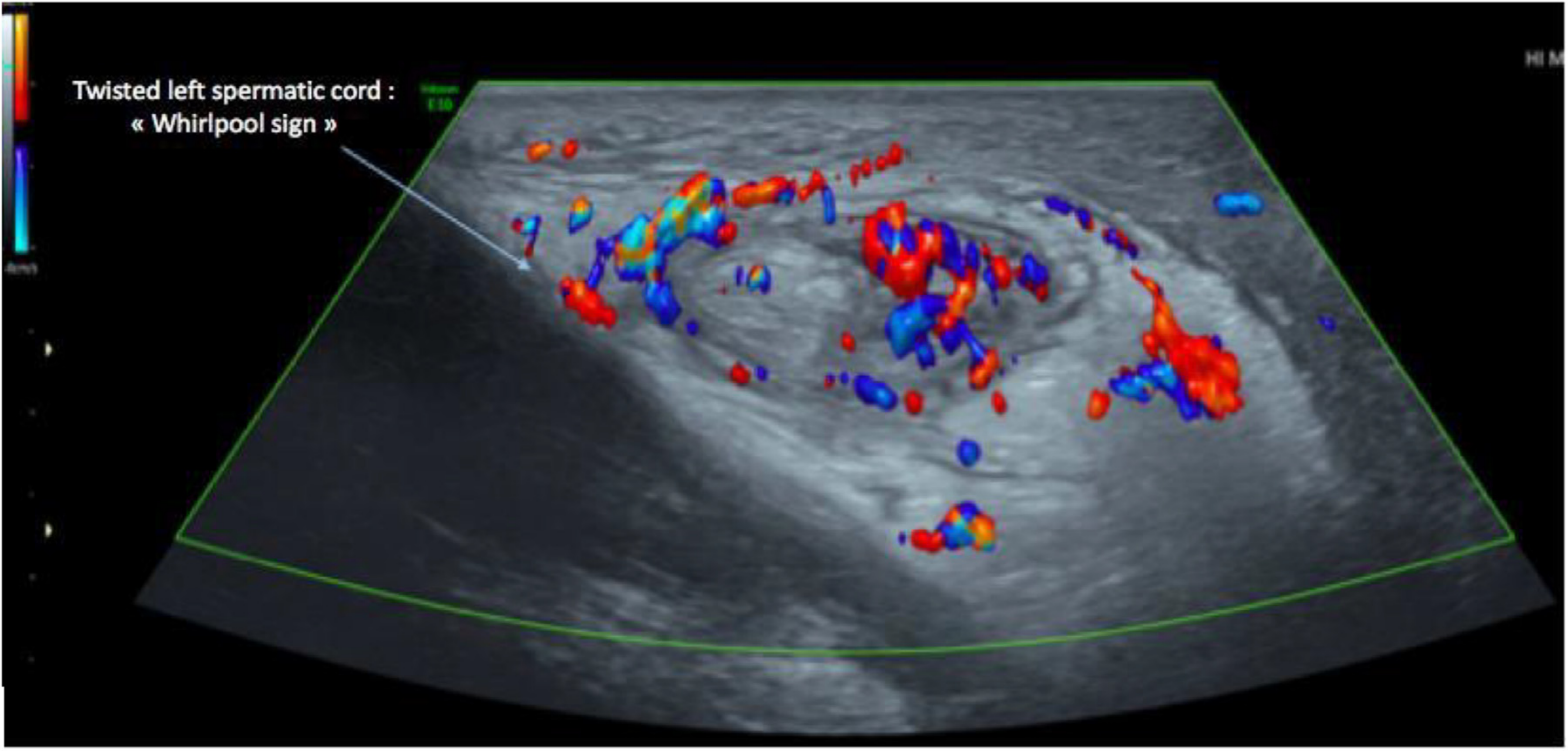
Study of the left spermatic cord in Doppler mode: presence of torsion with an image of a “Whirlpool sign” turn.

The ultrasound study was performed on both B-mode and Doppler modes. The findings revealed an ascended left testicle that appeared heterogeneous and hypoechoic. Small hypoechoic zones were also observed within the testicle (as shown in Fig. 2). In Doppler study, there was no evidence of blood flow in the left testicle (as depicted in Fig. 3, Fig. 4). Furthermore, the presence of a “Whirlpool sign” (as illustrated in Fig. 5) indicated torsion of the left spermatic cord.

**Fig. 6:**
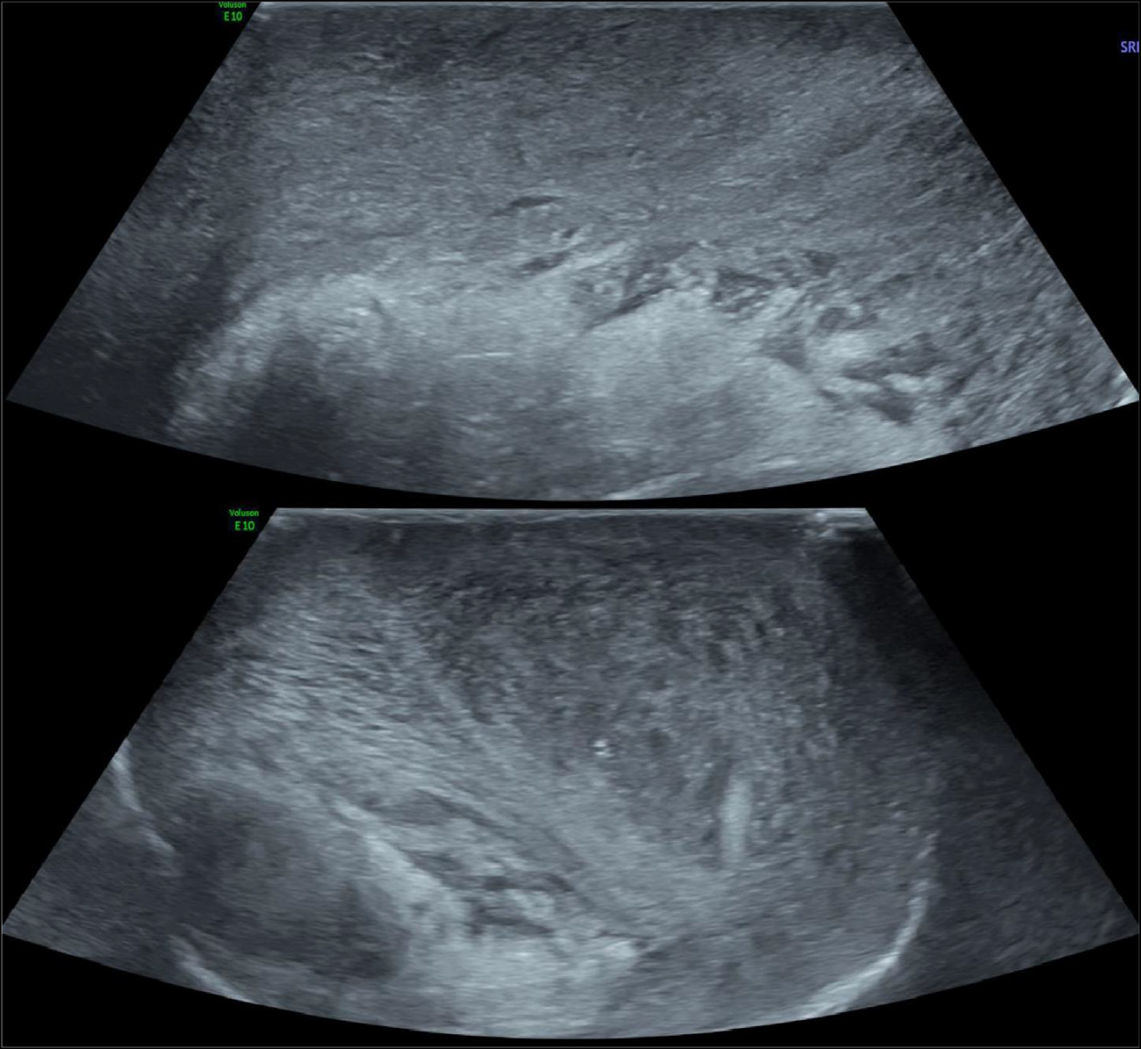
Hyperechoic thickening of the scrotal envelopes compatible with scrotal wall hematoma.

In addition, the ultrasound examination revealed a swollen and heterogeneous epididymal head, and a low abundance hyperechoic collection within the vaginalis tunica consistent with a hematocele (as seen in Fig. 2 ). Furthermore, thickening of the scrotal envelopes (hyperechoic) was observed, indicating the presence of a scrotal wall hematoma (Fig. 6).

**Fig. 7:**
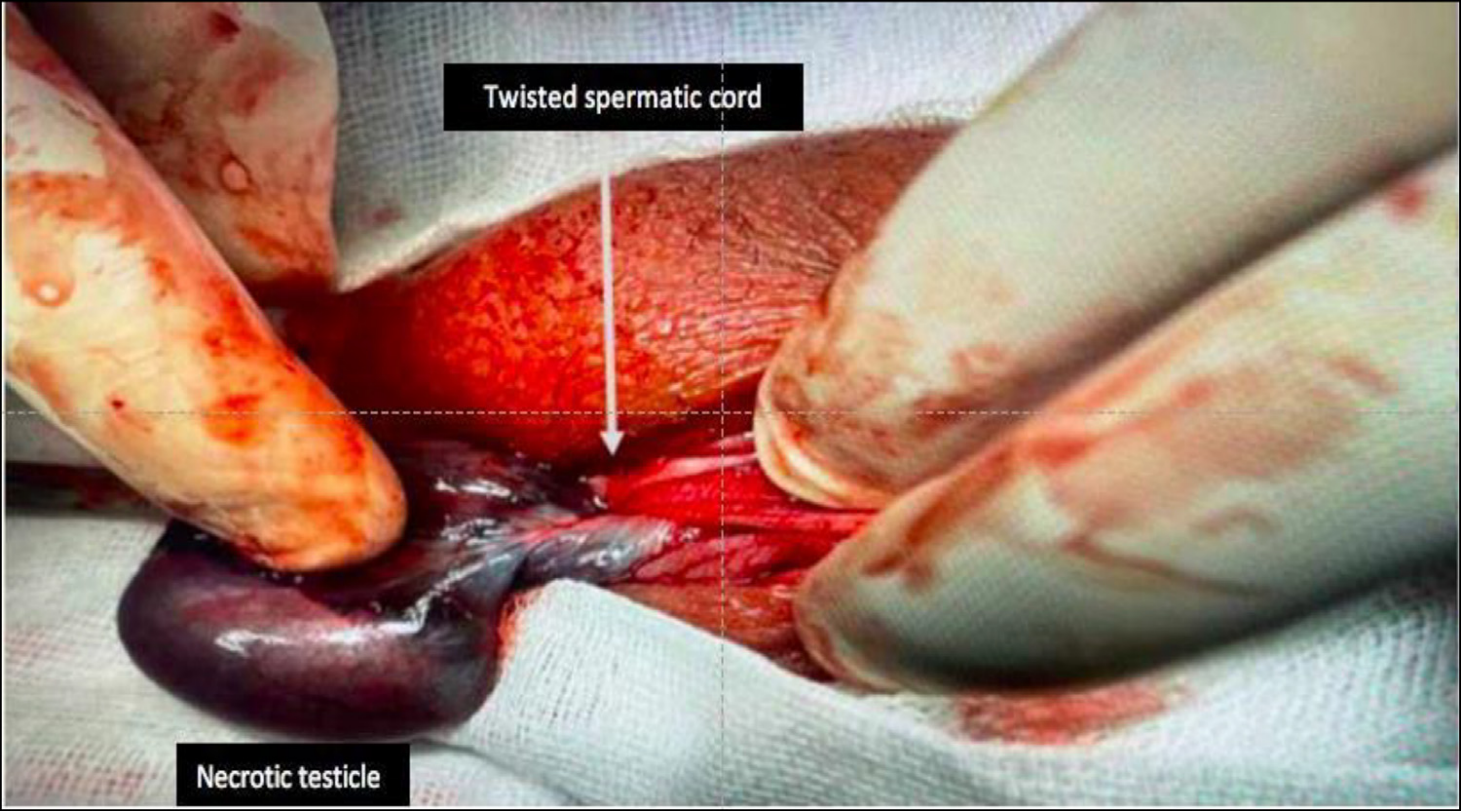
Surgical exploration: twisting of the left spermatic cord and rotation of left testicle with a necrotic blackish left testicle.

Surgical exploration of the scrotum revealed intravaginal torsion of the left testicle, with sero-sanguineous fluid in the vaginal sac. The color of the left testicle was blackish, with no change of coloration after untwisting the testicle and applying warm saline-soaked gauze to it (Fig. 7).

A left orchiectomy was performed with contralateral orchidopexy. The anatomo-pathological study of the operative specimen confirmed the operative results of testicular necrosis.

## Discussion

Testicular torsion is defined as the twisting of the spermatic cord and its contents.

It is the most common surgical emergency in urology, with orchidectomy occurring in 42% of men under the age of 25 who have undergone testicular torsion surgery. Trauma is an overlooked cause of this pathology leading to potential delays in appropriate treatment. Recognizing the association between trauma and the development of a testicular torsion is crucial to ensure timely and effective interventions, thereby avoiding potentially severe consequences and ensuring optimal patient outcomes [5,6].

The most important risk factor for testicular torsion is an anatomical predisposition, most commonly the so-called “bell clapper deformity,” corresponding to a high or complete impingement of the testis and spermatic cord by the vaginal sheath.

The cremaster muscle spirals around the spermatic cord, and its contraction has a rotating effect on the testis. Strong contraction of this muscle frequently observed in trauma, leads to rotation and then torsion of the susceptible, freely mobile testis [3,[7], [8], [9]].

Distinguishing clinically between torsion and acute traumatic injury can be, sometimes, very challenging due to the similarity in their clinical presentations, characterized by sudden scrotal pain and swelling.

If the clinical suspicion of testicular torsion is high, no further investigations are necessary, and immediate surgical evaluation is required. In cases where the physical examination is inconclusive, color Doppler ultrasonography is a valuable tool for diagnosing testicular torsion. It is considered the most effective imaging modality for evaluating patients suspected of having testicular torsion [10,11].

It facilitates the assessment of scrotal contents and provides definitive information on testicular structures, blood flow within scrotal structures, and the presence of intratesticular or extra-testicular hematomas and fluid accumulation [1,12].

Torsion of the testicle or torsion of the spermatic cord causes blockage of blood flow in the veins first and then in the arteries. The degree of testicular ischemia depends on the degree and duration of torsion [13].

Ultrasound may show a distinctive aspect with an initial homogeneous decrease in echogenicity of the test. In later stages, heterogeneous echogenicity is noted, with anechoic and cystic areas representing hemorrhagic infarction and focal tissue necrosis [14] (Fig. 2).

Doppler results are similar to those of a non-traumatic torsion. Arterial flow may be seen in a low-grade torsion. Spectral Doppler analysis of the intratesticular arteries, to identify high resistance flow compared to the contralateral testis, and evaluation of the spermatic cord by looking for the whirlpool sign (spermatic cord torsion) (Fig. 5), and redundant spermatic cord and epididymal head pseudomass (Fig. 2), are necessary to prevent false-negative investigations [15,16].

If an inconclusive finding is detected by an imaging technique, the patient must be recommended for urgent surgical exploration [17].

Orchiectomy is the terminal option in the management of testicular torsion, a necrotic and irrecoverable testis (Fig. 7) should be surgically removed [18].

## Conclusion

Post-traumatic testicular torsion is a well-documented but poorly appreciated entity. Physicians must always try to think and see beyond the obvious because the clinical presentation can sometimes be misleading. Thus, torsion pathology should always be considered in any post-traumatic acute scrotal pain.

In cases where there is suspicion of traumatic testicular torsion, conducting a thorough examination of the scrotum is crucial. When uncertainty persists, Doppler ultrasound is widely regarded as the “gold standard” for ruling out torsion in patients with blunt testicular injury.

Preserving the testicles is indeed the ultimate goal in cases of testicular torsion, and it relies on 2 key factors: the duration of symptoms and the promptness of treatment. Time is of the essence in testicular torsion, as the longer the duration of torsion, the higher the risk of testicular damage and necrosis.

## Ethics approval and consent to participate

Not applicable.

## Patient consent

Written informed consent was obtained from the child's parents, and legal guardian for publication of this case report and any accompanying images. A copy of the written consent is available for review by the Editor-in-Chief of this journal.

## Availability of data and materials

The data sets are generated on the data system of the CHU Hassan II of Fes, including the biological data and the interventional report.
